# Preparing tomorrow’s physicians for AI-driven healthcare: insights from a study on medical students’, interns’, and residents’ knowledge, attitudes, and educational needs

**DOI:** 10.3389/fmed.2026.1799061

**Published:** 2026-06-02

**Authors:** Asma F. Syeda, Fatima Alriyami, Asma Alshebli, Maha Alketbi, Azhar Rahma, Mohammed Al-Houqani

**Affiliations:** 1 National Institute for Health Specialties, United Arab Emirates University, Al Ain, United Arab Emirates; 2 College of Medicine and Health Sciences, United Arab Emirates University, Al Ain, United Arab Emirates

**Keywords:** AI readiness, artificial intelligence in healthcare, clinical decision support, ethical governance, medical education, mixed-methods study, trainee perceptions

## Abstract

**Background:**

Artificial intelligence (AI) is increasingly embedded in healthcare delivery, influencing clinical practice, medical education, and research. Despite rapid technological advancement, limited empirical evidence exists on how medical trainees across training levels perceive AI, their readiness to use it, and the educational, ethical, and system-level conditions required for responsible AI integration.

**Objective:**

This study aimed to examine medical and dental trainees’ knowledge, attitudes, real-world experiences, and educational needs related to AI in healthcare, with particular attention to trust, workflow integration, human oversight, and institutional governance.

**Methods:**

An explanatory sequential mixed-methods design was employed. A structured online survey was distributed to undergraduate and postgraduate medical and dental trainees across the United Arab Emirates (UAE) (*n* = 154). Quantitative data were analyzed descriptively. Semi-structured interviews were conducted with a purposive subsample of participants (*n* = 16) and analyzed using inductive reflexive thematic analysis following Braun and Clarke’s framework. Open-ended survey responses were used to support triangulation. Integration of findings was achieved through a joint display linking quantitative results with qualitative themes and illustrative data.

**Results:**

Survey responses (*n* = 154) demonstrated moderate awareness of AI applications, limited formal AI training, and strong support for structured AI education. Qualitative interviews (*n* = 16) revealed four overarching themes: (1) perceived value and normalization of AI, (2) AI as a tool for clinical support and workflow optimization, (3) trust, confidence, and human oversight, and (4) ethical, educational, and system-level preconditions. Trainees viewed AI as an assistive and time-saving tool for learning, research, and selected clinical tasks, while expressing context-dependent trust, emphasizing routine verification, human judgment, and concerns related to accuracy, bias, data security, accountability, and preservation of empathy in patient care.

**Conclusion:**

Trainees recognize the transformative potential of AI but emphasize the need for longitudinal, clinically relevant education, robust ethical governance, institutional support, and continued human oversight. These findings suggest that preparing future physicians for AI-enabled healthcare may require human-centered, ethically grounded, and system-ready approaches that align education, workflow integration, and governance.

## Introduction and background

Artificial intelligence (AI) has emerged as a transformative force in healthcare, with applications spanning diagnostics, imaging, workflow optimization, research, and medical education (1, 2). Advances in machine learning, natural language processing, and large language models have expanded AI’s capacity to support clinical decision-making, automate documentation, and enhance academic productivity (3, 4). AI has the potential to reduce administrative and documentation burdens, which may allow clinicians to spend more time with patients and strengthen compassionate, patient-centered care, rather than detract from the clinician-patient relationship (5). As healthcare systems increasingly adopt AI-enabled tools, physicians are expected to interact with these technologies as part of routine clinical practice (6).

Medical education has been slower to systematically integrate AI training into curricula, often leaving trainees to adopt AI tools informally and without structured guidance (7). Previous studies have highlighted gaps in AI literacy, uncertainty regarding appropriate use, and strong trainee interest in formal education focused on clinical relevance, ethical appraisal, and safe implementation (3, 7, 8).

Recent literature has emphasized the growing importance of preparing future physicians for AI-enabled healthcare. Multiple reviews and position papers highlight the need for structured AI education that encompasses foundational concepts, ethical considerations, interpretability, and clinical application (3, 6, 9). These studies consistently report strong learner interest in AI training and advocate for competency-based curricula integrated into undergraduate and postgraduate medical education. However, much of the existing literature remains conceptual or review-based, offering curricular frameworks and competency lists without sufficient empirical evidence drawn from trainees’ lived experiences.

Several systematic and narrative reviews have examined AI integration in medical education, identifying persistent challenges such as lack of validated curricula, insufficient faculty expertise, ethical concerns, and infrastructure limitations (3, 5, 9). While these reviews provide valuable global overviews, they often aggregate heterogeneous studies and lack contextualized insights into how trainees actually use AI tools in academic and clinical settings. Moreover, most studies focus predominantly on medical students, with limited attention to interns and residents who operate in high-stakes clinical environments and face different expectations, responsibilities, and time pressures.

Emerging empirical studies suggest that trainees’ trust in AI is highly context dependent. Learners report greater acceptance of AI for low-stakes academic tasks such as studying, summarization, and exam preparation, while expressing reservations about its use in high-stakes clinical decision-making (6). Concerns regarding accuracy, bias, transparency, data security, and professional accountability are recurrent themes across the literature. Clinicians are more likely to use AI tools when they are useful, easy to fit into daily work, easy to understand, and well governed. However, how these factors apply to early clinical training has not been well studied (10). Notably, several authors emphasize that AI may function as an assistive tool rather than an autonomous decision-maker, reinforcing the continued centrality of human judgment, empathy, and clinician accountability in patient care.

Despite recognition of clinical/AI decision support integration barriers, there remains limited research on how trainees’ perceptions of institutional and governmental roles—including expectations for policy development, ethical oversight, and the provision of safe, validated AI tools—influence readiness for AI integration. Existing studies on clinician access to AI and decision support systems highlight practical and interface challenges but do not investigate how trainees anticipate engaging with these systems or how educational and institutional systems can better prepare them (11).

Furthermore, much of the existing literature originates from Western contexts, limiting its applicability to other healthcare systems with distinct regulatory frameworks, educational structures, and digital health strategies. There is a particular scarcity of mixed-methods research capturing trainee perspectives within Middle Eastern healthcare systems, where national digital health initiatives coexist with evolving educational and regulatory landscapes.

To address these gaps, this study uses an explanatory sequential mixed methods design to examine medical and dental trainees’ knowledge, attitudes, experiences, and training needs related to AI in healthcare in the United Arab Emirates. By combining survey data with in-depth interviews across training levels, the study explores how trainees use and perceive AI in academic, clinical, and research settings, while also examining system-level factors such as institutional policies, ethical governance, IT support, and expectations of government involvement. These findings provide context-specific empirical insights that may inform curriculum development, institutional policy, and national strategies for ethical, human-centered AI integration in healthcare.

## Materials and methods

### Study design

This study employed an explanatory sequential mixed-methods design, in which quantitative survey data were first collected and analyzed, followed by qualitative semi-structured interviews to explain and contextualize the quantitative findings. This design enabled both broad characterization of trainee perspectives and in-depth exploration of underlying experiences and interpretations.

### Setting and participants

The study was conducted across multiple healthcare and educational institutions in the United Arab Emirates (UAE), including undergraduate medical students from United Arab Emirates University (UAEU) and postgraduate trainees (interns and residents) enrolled in accredited medical and dental training programs across Abu Dhabi, Dubai, Sharjah and Ajman.

As part of national accreditation and training oversight processes, centralized administrative records of trainee contact information were available, allowing identification of an accessible sampling frame for survey distribution.

### Quantitative phase

#### Survey instrument development

A structured, self-administered questionnaire was developed to assess trainees’ (medical students, interns, and residents) knowledge, awareness, attitudes, experiences, and educational needs related to artificial intelligence (AI) in healthcare.

Survey development followed established best practices in medical education research (12, 13). Items were informed by a review of relevant literature and adapted from previously published instruments assessing AI readiness, knowledge, attitudes, and practices among medical students and healthcare trainees (14–17). The questionnaire was further refined through multidisciplinary input from medical educators, clinicians, and AI researchers to ensure content validity, clarity, and relevance to the local educational context. Based on this expert feedback, item wording was iteratively revised prior to deployment.

Although formal cognitive interviewing and pilot testing were not conducted, the expert review process supported the face and content validity of the instrument. The final questionnaire included multiple-choice questions, Likert-scale items, and optional open-ended responses (Supplementary Data 1).

Key constructs were defined and measured as follows:

AI experience (self-reported knowledge level): Assessed using a single self-reported item with predefined categories (None, Basic, Intermediate, Advanced), reflecting perceived familiarity and engagement with AI rather than objectively measured competence.

AI awareness: Assessed using categorical items (Yes/No/Not sure) evaluating recognition of AI applications in healthcare.

Preparedness to work with AI: Measured using Likert-type items assessing perceived readiness to engage with AI in healthcare settings. These reflect subjective confidence rather than objectively assessed capability.

Privacy and data-sharing awareness: Assessed using multiple-response items identifying types of information considered unsafe to share with AI tools, including direct identifiers (e.g., patient name, ID) and indirect identifiers (e.g., clinical notes, imaging metadata). Participants could select multiple options, including “None of the above,” enabling identification of both correct understanding and misconceptions.

Open-ended survey responses were imported into NVivo and coded alongside interview data to support triangulation, contributing to the confirmation and refinement of qualitative themes.

Importantly, key constructs such as knowledge, preparedness, and attitudes toward AI are inherently complex and multifaceted. These constructs were assessed using self-reported survey items, which provide an approximate representation of trainees’ perceived competencies, experiences, and understanding.

#### Sampling and data collection

The survey was administered using REDCap and distributed via official institutional email lists to all trainees within the defined sampling frame. A total of 2,151 trainees were invited to participate.

Automated weekly reminders were sent to non-respondents using REDCap’s reminder functionality. In addition, the research team conducted supplementary follow-up through trainee communication channels (e.g., WhatsApp) to encourage participation.

Data collection occurred over a 4-month period (May–September 2025). A total of 154 trainees completed the survey, yielding a response rate of approximately 7.0%.

#### Quantitative data analysis

Quantitative data were analyzed using descriptive statistics (frequencies and proportions) using SPSS, Microsoft Excel, and Power BI.

Given the exploratory aims, convenience sampling, unequal subgroup sizes, and modest response rate, inferential statistical testing was not performed. The analysis focuses on describing observed patterns within the sample rather than making population-level inferences.

With a sample size of 154, the study provides reasonable precision for descriptive estimates (approximately ± 8% margin of error at the 95% confidence level for proportions near 50%).

### Qualitative phase

#### Sampling and recruitment

At the end of the survey, respondents were invited to indicate their willingness to participate in a follow-up interview. Of the 154 survey respondents, 45 expressed interest in qualitative participation. From this pool, participants were purposively selected using a maximum variation sampling strategy to ensure representation across training level (medical students, interns, and residents), gender, clinical exposure, and geographic/institutional setting.

Potential participants were contacted via phone to arrange interviews. Of the 45 individuals who initially expressed interest, 16 completed the interviews, 19 declined participation due to time or work commitments, and the remaining individuals were not reachable despite follow-up attempts.

The final interview sample comprised 16 participants, including 68.75% female and 31.25% male trainees. A total of 16 interviewees from three groups included residents (10), interns (4), Medical students from UAEU (2). Participants represented three training groups: postgraduate residents (62.50%, including trainees from R2–R5 and one recently completed resident), interns (25.00%), and undergraduate medical students (12.50%). Participants were based across multiple emirates, including Abu Dhabi, Dubai, Sharjah, and Ajman, reflecting geographic diversity within the UAE national training system.

**Table:** 

Interviewee	Current workplace (if applicable)	Training level	Year of study
Interview 1	Sharjah	Resident	Year 4
Interview 2	Abu Dhabi	Resident	Year 5
Interview 3	Abu Dhabi	Resident	Year
Interview 4	Dubai	Resident	Year 2
Interview 5	(Not mentioned)	Resident	Year
Interview 6	Dubai	Resident	Year 5
Interview 7	Sharjah	Resident	Year
Interview 8	Abu Dhabi	Resident	Year
Interview 9	Dubai	Resident	Year
Interview 10	Sharjah	Graduated from residency program	Year 4
Interview 11	Ajman	Intern	Intern
Interview 12	Dubai	Intern	Intern
Interview 13	Abu Dhabi	Intern	Intern
Interview 14	Abu Dhabi	Intern	Intern
Interview 15	Al Ain, UAE University	Medical student	Medical student
Interview 16	Al Ain, UAE University	Medical student	Medical student

This sampling approach resulted in a heterogeneous group with variation in training level, institutional affiliation, and clinical exposure, enabling the capture of diverse perspectives. However, as participation was voluntary, it is possible that individuals with greater interest in or exposure to AI were more likely to participate, and perspectives of less engaged or more skeptical trainees may be underrepresented.

#### Data collection

Semi-structured interviews were conducted online using an interview guide developed based on preliminary survey findings and relevant literature (Supplementary Data 2). The guide explored trainees’ experiences with AI, perceived benefits and risks, trust and verification practices, educational needs, and expectations regarding institutional and governmental roles. Interviews were audio-recorded, transcribed verbatim, and anonymized prior to analysis.

#### Qualitative data analysis

Qualitative data were analyzed using inductive reflexive thematic analysis following Braun and Clarke’s six-phase framework (18). NVivo software was used to support data organization, coding, and retrieval, and to facilitate the development and iterative refinement of the coding framework (codebook).

Analysis was conducted iteratively and involved:

- Familiarization with the data
- Generation of initial codes
- Development of candidate themes
- Review and refinement of themes
- Definition and naming of themes
- Integration and interpretation of findings

Coding was conducted collaboratively by members of the research team. Initial coding was performed independently by multiple researchers, followed by iterative discussions to compare interpretations, refine codes, and develop themes. Discrepancies in coding and theme interpretation were resolved through discussion and consensus. A coding framework was developed and continuously refined throughout the analytic process to ensure coherence and consistency.

Reflexivity was addressed through regular team discussions, during which researchers reflected on their disciplinary backgrounds, clinical roles, and prior assumptions regarding AI in healthcare, and how these perspectives might influence data interpretation. Analytic decisions and theme development were documented through iterative discussions.

To enhance rigor and credibility, findings were developed through ongoing engagement with the data and team-based interpretation. Open-ended survey responses were imported into NVivo and coded alongside interview transcripts using the same coding framework. These responses were used to support triangulation by confirming, enriching, and refining themes identified from the interview data, rather than generating a separate thematic structure.

The qualitative component was conducted and reported in accordance with the consolidated criteria for reporting qualitative research (COREQ), with the completed checklist provided as Supplementary Data 3.

### Integration of quantitative and qualitative data

The qualitative phase was designed to explain and expand upon key quantitative findings. Interview data were used to explore underlying reasons for observed patterns in AI awareness, preparedness, trust, and educational needs.

Integration occurred at the interpretation stage, where qualitative themes were used to contextualize quantitative trends and highlight areas of convergence and divergence. Open-ended survey responses were used to support triangulation and integrated into the coding process to provide additional depth and breadth to the qualitative findings.

### Use of artificial intelligence tools

AI-assisted tools (e.g., ChatGPT) were used solely for language editing and formatting purposes during manuscript preparation. No AI tools were used in the study design, data collection, data analysis, coding, or interpretation of findings. All scientific content, analyses, and conclusions were independently developed and verified by the authors.

Furthermore, we ensured that no identifiable or sensitive data were entered into any AI systems at any stage of the study.

### Ethical approval

The study was approved by the Social Sciences Ethics Committee of United Arab Emirates University (institutional review board approval ERSC_2025_6285). All participants received an information sheet describing the study objectives and procedures, and access to the survey was provided only after informed consent was obtained. The study was conducted in accordance with local legislation and institutional requirements.

## Results—quantitative analysis

Given the exploratory and descriptive nature of this study, results are presented using summary statistics without inferential testing. Observed differences across trainee groups represent descriptive patterns within the sample and should be interpreted cautiously, particularly given the unequal group sizes.

### Participant characteristics

A total of 154 trainees participated in the survey, including interns (*n* = 11), medical students (*n* = 17), and residents (*n* = 126), reflecting a predominance of postgraduate trainees within the sample.

Participants represented a range of training levels. Among undergraduate trainees, the largest proportion was in Year 3 (29.41%, *n* = 5) followed by other years.

Among postgraduate trainees, the largest proportions were in Year 2 (27%, *n* = 34) and Year 1 (23.8%, *n* = 30), followed by Year 3 (23.0%, *n* = 29) and Year 4 (20.6%, *n* = 26). Smaller proportions were observed in Year 5 (4.0%, *n* = 5). Interns (*n* = 11) represented the transitional stage between undergraduate and postgraduate training.

The sample was predominantly female, with 72.1% (111/154) female participants and 27.9% (43/154) male participants. Gender distribution varied across training groups, with medical students comprising 94.1% female (16/17), residents 67.5% female (85/126), and interns 90.9% female (10/11).

Overall, the sample included trainees across multiple levels of training, enabling descriptive comparison across the training continuum. Respondents were drawn from multiple healthcare institutions across the UAE, reflecting geographic and institutional diversity.

### Knowledge and awareness of artificial intelligence

Conceptual understanding of artificial intelligence: Overall, trainees demonstrated a strong conceptual understanding of artificial intelligence (AI). The majority correctly identified AI as “computer systems that simulate human intelligence” (87.0%, 134/154), with consistently high recognition across trainee groups, including 90.9% of interns, 76.5% of medical students, and 88.1% of residents.

Incorrect or uncertain responses were relatively uncommon. A small proportion selected alternative definitions, including software for hospital logistics (6.5%), while 4.5% reported being unsure. Only 1.9% selected the incorrect definition of AI as a robot performing surgery.

Experience with AI: Overall, most trainees reported basic experience with AI (63.64%, *n* = 98), followed by intermediate experience (28.6%, *n* = 44). Only a small proportion reported advanced experience (2%, *n* = 3), while 5.8% (*n* = 9) reported no prior experience.

When examined across training years, basic experience remained the most common level across all trainee groups, including interns, medical students, and residents while advanced experience was rare throughout as shown in Table 1.

**TABLE 1:** Experience with AI across all trainee groups and training years.

Trainee group/year	Advanced	Basic	Intermediate	None	Total
Internship		54.5% (6)	27.3% (3)	18.2% (2)	11
Internship year		54.5% (6)	27.3% (3)	18.2% (2)	11
Medical students		58.8% (10)	41.2% (7)		17
Year 1		50.0% (1)	50.0% (1)		2
Year 2		33.3% (1)	66.7% (2)		3
Year 3		80.0% (4)	20.0% (1)		5
Year 4		66.7% (2)	33.3% (1)		3
Year 5		50.0% (1)	50.0% (1)		2
Year 6		50.0% (1)	50.0% (1)		2
Residents	2.38% (3)	65.08% (82)	26.98% (34)	5.56% (7)	126
Year 1	3.33% (1)	66.67% (20)	20.00% (6)	10.00% (3)	30
Year 2	2.94% (1)	67.65% (23)	29.41% (10)		34
Year 3	3.45% (1)	55.17% (16)	31.03% (9)	10.34% (3)	29
Year 4		76.92% (20)	23.08% (6)	26
Year 5		42.86% (3)	42.86% (3)	14.29% (1)	7
Total	2.0% (3)	63.6% (98)	28.6% (44)	5.8% (9)	154

Awareness of AI applications in healthcare: Despite strong conceptual understanding, awareness of AI applications in healthcare was more variable. Overall, 51.9% of participants (80/154) reported being aware of AI applications, while 27.9% (43/154) were unsure and 20.1% (31/154) reported no awareness.

Awareness appeared higher among earlier-stage trainees, with 72.7% of interns and 70.6% of medical students reporting awareness, compared to 47.6% of residents. In contrast, uncertainty was more frequently reported among residents (31.7%) than among interns (18.2%) and medical students (5.9%).

Perceived applications of AI in healthcare: Participants most identified AI applications related to diagnostic support and workflow efficiency. Overall, diagnosing diseases (44.2%) and automating documentation (43.5%) were the most frequently selected applications, followed by predicting disease outbreaks (37.0%).

Diagnosing diseases: 44.2% overall
  Interns: 72.7% (8/11)
  Medical students: 64.7% (11/17)
  Residents: 38.9% (49/126)
• Automating documentation: 43.5% overall
  ○ Interns: 63.6% (7/11)
  ○ Medical students: 58.8% (10/17)
  ○ Residents: 39.7% (50/126)
• Predicting outbreaks: 37.0% overall
  ○ Interns: 54.5% (6/11)
  ○ Medical students: 52.9% (9/17)
  ○ Residents: 33.3% (42/126)

Very few participants believed AI would replace doctors completely (0.6% overall).

### Perceived preparedness for AI use

Perceived preparedness to work with AI in healthcare varied across trainee groups. Overall, 61.0% of participants (94/154) reported feeling prepared, while 23.4% (36/154) were unsure and 14.9% (23/154) reported not feeling prepared.

Preparedness appeared higher among interns (81.8%, 9/11) compared to residents (61.9%, 78/126) and medical students (41.2%, 7/17). In contrast, uncertainty was more commonly reported among medical students (52.9%, 9/17) and residents (21.4%, 27/126), while no interns selected the “not sure” option.

Exposure to AI training: Most trainees reported no prior AI training (70.1%, 108/154). Formal AI training was limited, reported by 12.3% of participants (19/154). This included 27.3% of interns (3/11), 11.8% of medical students (2/17), and 11.1% of residents (14/126).

A slightly higher proportion reported informal exposure to AI training, with 17.5% of participants (27/154) indicating informal learning. This was more common among medical students (23.5%, 4/17) and residents (17.5%, 22/126), compared to 9.1% of interns (1/11).

Across all trainee groups, the majority reported no prior AI training, although small proportions indicated either formal or informal exposure. Overall, these findings suggest that structured AI training remains limited across the training continuum, with most trainees relying on informal or self-directed learning where exposure exists. These observations represent descriptive patterns within the sample and should be interpreted cautiously.

Need for AI training in medical curriculum: There was strong support for incorporating AI training into the medical curriculum. Overall, 43.5% of participants (67/154) preferred AI training to be required, followed by 27.3% (42/154) favoring workshop-based training, and 24.7% (38/154) preferring elective options. Only a small proportion (3.9%, 6/154) indicated no interest in AI training.

Preferred learning formats: Preferences for learning formats reflected a consistent emphasis on applied and interactive approaches.

Workshops were the most preferred format, selected by 32.5% of responses overall, followed by simulations (27.6%) and online modules (19.6%). Traditional formats such as lectures (10.3%) and guest speakers (10.1%) were less frequently selected.

Across trainee groups, interns showed relatively higher preference for simulation-based learning (37.0%), while residents demonstrated a strong preference for workshops (33.3%). Preferences among medical students were more evenly distributed across formats.

Topics of interest in AI education: Participants expressed interest in a range of AI-related topics, with emphasis on clinically relevant applications.

The most selected topic was clinical use cases (34.2%), followed by evaluating AI tools (26.2%), machine learning basics (19.9%), and ethics and law (18.7%). Very few participants selected “other” topics (1.0%).

Interest patterns were broadly consistent across trainee groups, with clinical applications and practical evaluation skills emerging as priority areas.

### Trainee attitude toward AI in healthcare

Overall attitudes toward AI in healthcare: A large majority of participants agreed that AI could improve diagnostic accuracy, with consistently high agreement across groups, including 77.8% (98/126) of residents, 82.3% (14/17) of medical students, and 63.7% (7/11) of interns (combined agree and strongly agree). Similarly, there was strong support for physician involvement in AI development, reported by 81.8% of residents (103/126), 88.2% of medical students (15/17), and 81.8% of interns (9/11).

There was also broad agreement that AI may be integrated into medical education. Agreement was reported by 76.2% of residents (96/126), 82.4% of medical students (14/17), and 81.8% of interns (9/11).

Perceptions of AI and the human role in medicine: Across all trainee groups, participants strongly endorsed the view that AI cannot replace human aspects of care. Agreement with the statement “AI cannot replace human empathy” was reported by 78.9% of residents (99/126), 82.3% of medical students (14/17), and 90.9% of interns (10/11). There was strong agreement among participants that physicians should be involved in AI development. Agreement with this statement was reported by 81.8% of residents (103/126), 88.2% of medical students (15/17), and 81.8% of interns (9/11).

Perceptions of AI risks and ethical considerations: Trainees demonstrated awareness of ethical concerns related to AI, although responses varied across groups. Agreement that AI raises important ethical concerns was reported by 69.8% of residents, 76.5% of medical students, and 54.5% of interns, with a notable proportion of participants in each group also selecting neutral responses.

Perceptions of AI-related bias and system limitations were mixed. While some participants expressed confidence in AI systems, a substantial proportion particularly among interns and residents reported uncertainty regarding how AI operates in clinical settings, with 45.6% of residents (57/126) and 63.6% of interns (7/11) selecting neutral responses to statements regarding understanding of AI in clinical practice.

Perceptions of job security and professional impact: Views regarding the impact of AI on physicians’ job security were more divided. A greater proportion of residents expressed concern, with 22.3% (28/126) (agree/strongly agree) indicating that AI threatens job security, compared to 11.8% of medical students (2/17) and 18.2% of interns (2/11). However, a substantial proportion across all groups disagreed or remained neutral, suggesting uncertainty rather than consensus on this issue.

Interest in AI learning and engagement: Interest in further learning about AI was high across all trainee groups. Agreement with the statement “I am interested in learning more about AI” was reported by 84.9% of residents (107/126), 82.4% of medical students (14/17), and 72.7% of interns (8/11).

Overall, trainees demonstrated positive attitudes toward the clinical potential of AI and strong support for its integration into medical education, while also expressing uncertainty regarding ethical implications, system functioning, and professional impact.

### AI use and clinical exposure

Patterns of AI use across academic and clinical contexts: Use of artificial intelligence (AI) tools varied across academic and clinical activities, with consistently higher engagement in learning-related tasks compared to direct clinical applications.

Across all trainee groups, AI was most frequently used for understanding complex topics and academic support activities. Among residents, 36.3% (46/126) reported sometimes and 32.3% (41/126) reported often using AI to generate explanations, while 26.6% (34/126) reported sometimes and 37.1% (47/126) reported often using AI for summarizing medical content.

Medical students demonstrated high engagement with AI for learning tasks, with 47.1% (8/17) reporting very often use for generating explanations and 35.3% (6/17) very often use for summarizing content. Interns showed similar patterns, with 54.5% (6/11) reporting sometimes and 36.4% (4/11) often using AI for understanding complex topics.

Use of AI for assessment and academic tasks: AI tools were also used for practice-based learning activities, such as generating multiple-choice questions (MCQs). Among residents, 33.1% (42/126) reported sometimes use and 19.4% (24/126) often use.

Medical students showed relatively higher engagement, with 41.2% (7/17) reporting sometimes and 23.5% (4/17) very often use, while interns demonstrated more variable patterns.

Similarly, AI-assisted writing tasks were commonly reported, particularly among medical students, where 47.1% (8/17) reported sometimes use, compared to 35.5% (45/126) of residents and 45.5% (5/11) of interns.

Clinical use of AI tools: In contrast, the use of AI in clinical contexts was less frequent across all groups.

Use of clinical decision support systems incorporating AI was limited, with 33.9% of residents (43/126) and 41.2% of medical students (7/17) reporting never using such systems. Among interns, most reported occasional use, with 63.6% (7/11) indicating sometimes use.

Exposure to AI-supported diagnostic tools during clinical rotations was similarly limited. Most medical students (58.8%, 10/17) and a substantial proportion of residents (31.5%, 40/126) reported no exposure, while interns primarily reported occasional exposure (45.5%, 5/11 sometimes).

Use of AI for differential diagnosis or investigation suggestions was generally occasional, with 32.3% of residents (41/126), 52.9% of medical students (9/17), and 45.5% of interns (5/11) reporting sometimes use.

Administrative and organizational use: Use of AI for administrative tasks, such as managing academic or clinical schedules, was moderate. Among residents, 29.0% (37/126) reported sometimes use, while 29.4% (5/17) of medical students and 45.5% (5/11) of interns reported similar patterns.

Overall, these findings suggest a distinction between academic and clinical use of AI tools. Trainees across all groups reported frequent use of AI for learning and academic productivity, whereas clinical integration and exposure remain limited and inconsistent.

### Privacy and data sharing

Participants were asked to identify types of information that should not be shared with general AI tools, allowing multiple selections.

Across all trainee groups, there was strong recognition of direct identifiers as sensitive information. The majority correctly identified patient identifiers (e.g., Patient ID) as inappropriate for sharing, including 81.8% of interns, 88.2% of medical students, and 83.3% of residents.

Similarly, a high proportion of participants identified patient-related clinical information (Patient Name) as sensitive, with 90.9% of interns, 82.4% of medical students, and 84.1% of residents selecting this option.

In contrast, awareness of less obvious or indirect identifiers was more variable. Recognition of clinical notes as sensitive information was lower, particularly among medical students (52.9%) and residents (54.8%), compared to interns (63.6%). Similarly, identification of imaging data as potentially sensitive was inconsistent, with 18.2% of interns, 41.2% of medical students, and 32.5% of residents selecting this option.

A notable proportion of participants selected “None of the above,” particularly among medical students (11.8%) and residents (9.5%), indicating potential misconceptions regarding safe data-sharing practices with AI tools.

## Results—qualitative analysis

**FIGURE 1:**
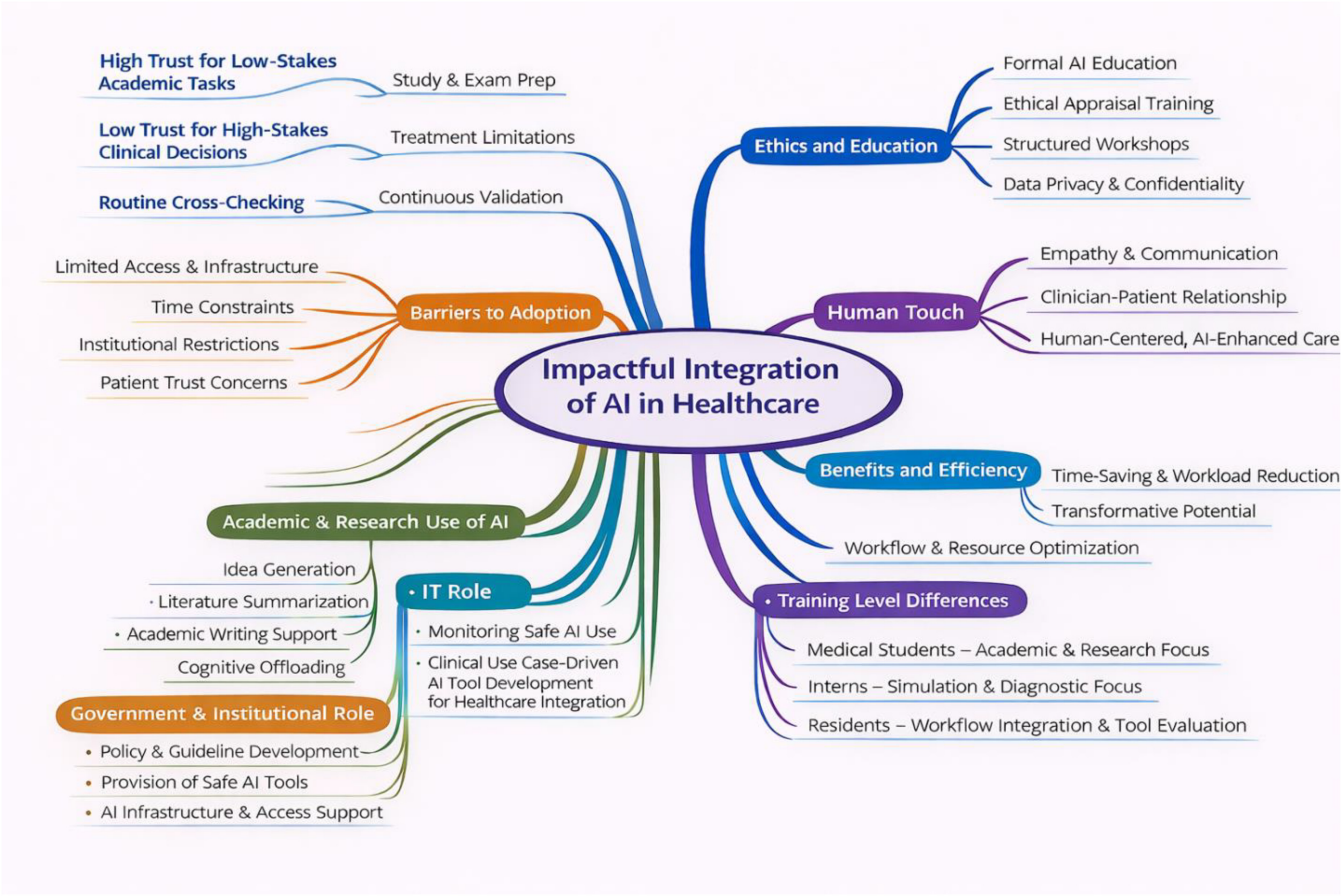
Conceptual mind map illustrating the interrelationships among key themes influencing the integration of artificial intelligence in healthcare training, derived from qualitative thematic analysis.

Qualitative data were obtained from semi-structured interviews (*n* = 16) and open-ended survey responses. Both data sources were analyzed using a unified thematic framework. An expanded thematic framework with illustrative quotations is provided in Supplementary Data 4. A conceptual mind map summarizing the relationships among the main themes and sub-themes is presented in Figure 1.

### Main theme 1: perceived value and normalization of AI

Definition: AI is viewed as a beneficial, timesaving, and increasingly embedded tool in learning, research, and clinical workflows.

1.1 Optimism and enthusiasm toward AI: Participants described AI as an efficient and potentially transformative tool in healthcare, particularly highlighting its ability to save time and reduce workload by supporting documentation and routine tasks. Key perceived benefits included:

- AI as efficient, exciting, and transformative
- Time-saving and workload reduction

“AI is going to have a great impact on healthcare. It will make our work easier and faster, but it should be used in the right way.” (Source: Interview 3—Dental Intern)

“AI can save a lot of time for doctors because it helps with documentation and routine work, so the doctor can focus more on the patient.” (Source: Interview 11—Medical student/resident)

1.2 Routine use of AI for academic productivity: Many trainees reported daily or frequent use of AI as part of their study routines, for tasks such as studying, summarization, generating practice MCQs, and exam preparation. AI was described as part of their routine workflow:

- Daily or frequent use
- Studying, summarizing, MCQs, exam preparation
- AI as part of routine workflow

“I use ChatGPT and open evidence almost every day during the rounds if I want to look something up quickly.” (Source: Interview 16—Intern)

“I used it to generate several questions so that I can train myself for the question material when studying for exams.” (Source: Medical student interview)

“Instead of looking things up manually, I just use AI as part of my daily workflow to save time.” (Source: Interview 6—Resident)

1.3 Integration of AI in academic and clinical research and scholarly work: Participants described a growing reliance on AI for research-related tasks, while maintaining awareness of its limitations. Common applications included:

- Research idea generation
- Literature summarization
- Academic writing support
- Cognitive offloading with verification

“I’ve used AI to generate ideas for research questions and even to create different types of questions when I’m preparing for board exams or presentations.” (Source: Interview 6—Senior prosthodontic resident)

“It helps me find evidence-based knowledge and gives concise information based on journals and medical reports.” (Source: Interview 3—Dental intern)

“I use AI to correct grammar mistakes and add vocabulary when I’m writing my research.” (Source: Interview 6—Senior prosthodontic resident)

“AI makes things faster, but I still have to recheck everything before I use it.” (Source: Interview 12—Resident, anesthesia)

These perceptions were echoed in open-ended survey responses, reinforcing trainees’ optimism regarding AI’s efficiency and potential benefits. For example, one survey respondent (Resident) noted: “*It excites me how it will be a time saver and will help decrease medical errors.*”

### Main theme 2: AI as a tool for clinical support and workflow optimization

Definition: AI is perceived to function as an assistive system that enhances diagnostic efficiency, workflow, and resource management without replacing clinicians.

2.1 Clinical decision support and diagnostic augmentation: Participants viewed AI as especially useful for supporting diagnostic tasks. They gave examples of AI aiding ECG, radiology, and imaging support, improving triage and prioritization, and enabling faster detection and early warnings of clinical issues:

- ECG, radiology, and imaging support
- Triage and prioritization
- Faster detection and early warnings

“With X-rays and CTs they have implemented AI. So we don’t really have to wait much for the radiologist report… it helps the assessment to be quicker and more efficient.” (Source: Interview 6—Emergency medicine resident)

“AI will be used best in diagnostic imaging… it will help us a lot and reduce the time needed.” (Source: Interview 10—Resident)

“There’s more [that] radiologists can do besides writing reports and pattern recognition which AI can do now.” (Source: Interview 16—Intern, radiology)

“It can give insights about organized data specifically in patient flow and how you manage high-risk or low-risk patients accordingly.” (Source: Interview 10—Resident)

“AI can help in registration areas and help prioritize patients.” (Source: Interview 10—Resident)

2.2 Workflow efficiency and resource optimization: Participants emphasized AI’s role in reducing administrative burden, improving documentation, and supporting workload distribution. They anticipated AI would provide:

- Reduced documentation burden
- Improved patient flow
- Administrative and operational support

“AI can help with routine stuff, for example writing notes… I would love to give the history verbally and the physical exam findings and it would just write the note for this patient for me.” (Source: Interview 16—Intern)

“Instead of the doctor taking more time doing documentation, it will be more focused on the patient itself.” (Source: Interview 11—Medical student)

“We used it to do the on-call rota… it’s a headache for a human being.” (Source: Interview 10—Resident)

“AI can organize schedules and administrative work that takes a lot of time.” (Source: Interview 9—Pediatric resident)

“AI can help with organizing data, schedules, and operational tasks.” (Source: Interview 9—Pediatric resident)

2.3 Breadth of AI tools used in practice and learning: Participants reported using a wide ecosystem of generative, educational, and clinical AI tools, reflecting increasing technological fluency. For example, interviewees mentioned using:

“ChatGPT, Perplexity, Claude for research.”

“Clinical Key AI, Anki, Uptodate AI clinical pathways.”

“Queen of Hearts — AI-driven ECG analysis tool for detecting acute occlusions.”

“ChatGPT—Amboss toggle for lecture topics I found challenging.” (Source: multiple interviews and open-ended survey responses.)

Survey responses further supported the perceived role of AI in reducing administrative burden and improving workflow efficiency. One survey respondent (Medical student) stated: “*Smoother workflow and less paperwork.*”

### Main theme 3: trust, confidence, and human oversight

Definition: This theme reflects a cautious use of AI, with participants emphasizing the need for verification, human judgment, and empathy.

3.1 Confidence gaps and perceived limitations: Participants consistently expressed concerns regarding AI accuracy and bias, questioned its reliability as a sole source of clinical information, and emphasized the irreplaceable role of human empathy and clinical judgment in patient care. Key concerns included:

- Concerns about accuracy and bias—Worries that AI may provide incorrect or biased information.
- Lack of empathy and clinical gestalt—Recognition that AI cannot replicate human empathy or intuitive clinical reasoning.
- Reliability issues—Uncertainty about depending on AI without human confirmation.

“AI will state the answer in a very professional way that makes you think this is the correct answer, but the knowledge, the background is wrong. That’s because it was fed in the wrong way.” (Source: Interview 12—Resident, Anesthesia)

“A robot can never show empathy just like humans… when a patient is going through a hard time and you sit and speak to them, I’m not sure how AI would do this. You can’t replace this at all.” (Source: Interview 6—Emergency Medicine Resident)

“I don’t rely on it 100% because it’s still new and it still has a lot of weakness. So I still rely on other sources like books or research pages.” (Source: Interview 8—Resident)

3.2 Context-dependent trust in AI: Participants demonstrated high trust in AI for low-stakes academic tasks, but maintained clear reservations regarding its use in high-stakes clinical decision-making. They consistently described routine cross-checking of AI outputs as an essential safeguard. They highlighted:

- High trust for low-stakes academic tasks—Willingness to use AI for studying and routine tasks.
- Low trust for high-stakes clinical decisions—Hesitation to rely on AI for critical patient care decisions.
- Routine cross-checking and validation—The practice of always verifying AI-generated information.

“I’ve been using AI during my studies mostly to help me generate questions when I’m studying for board preparation or exams, and it helps me a lot during presentations.” (Source: Interview 6—Senior prosthodontic resident)

“When it comes to treatments on their own, it’s still the clinician’s job. I can rely on AI to give me options, but not to a point where I would rely on it for the treatment itself.” (Source: Interview 2—Dental intern)

“I don’t rely on it fully… I still have to recheck after, especially in research and implant planning. It’s about 90% accurate, but there’s that 10% you always have to look after.” (Source: Interview 3—Dental intern)

3.3 Human oversight and professional responsibility: Participants consistently described AI as an assistive tool rather than an autonomous decision-maker, emphasized that final clinical decisions must remain with clinicians, and highlighted professional accountability as a non-transferable responsibility:

- AI as assistive, not autonomous—AI is a tool to aid, not replace, the clinician.
- Final decisions remain with clinicians—Clinicians must make the ultimate decisions.
- Accountability and professional judgment—Physicians remain accountable for outcomes and must apply their judgment.

“AI could answer complex clinical questions and help solve difficult scenarios faster, but it should not directly take decisions or place orders.” (Source: Interview 1—Resident)

“Diagnosis is amazing, but when it comes to treatment, it’s still the clinician’s job. AI can give suggestions, but execution should be by the doctor.” (Source: Interview 3—Dental Intern)

“If something goes wrong, who’s going to be responsible? Is it the AI, the doctor, or the institution? At the end of the day, it has to be the physician.” (Source: Interview 12—Resident, Anesthesia)

Open-ended survey comments similarly reflected trainees’ cautious approach to AI use and the importance of validation. For instance, a survey respondent (Resident) noted: “*AI can make mistakes and is not always accurate, so information must be checked.*”

### Main theme 4: ethical, educational, and system-level preconditions

Definition: This theme reflects participants’ recognition that effective AI integration may require ethical governance, structured education, institutional readiness, and clear boundaries to prevent misuse and harm.

4.1 Ethical, privacy, and governance concerns: Participants raised strong concerns regarding confidentiality, data security, bias, liability, and lack of transparency in AI systems. They emphasized issues of:

- Confidentiality and data security—protecting patient privacy when using AI.
- Bias and transparency—the risk of AI inheriting biases and the need for explainability.
- Liability and accountability—uncertainty about who is accountable for AI-driven decisions.

“You shouldn’t copy and paste any patient information into ChatGPT or any AI tool, because it’s private and confidential and it might leak to the public.” (Source: Interview 10—Urology resident)

“If you steer the AI in a certain direction, it will be biased and stick to that answer, even if it’s wrong.” (Source: Interview 10—Urology resident)

“If something goes wrong, who’s responsible? The AI, the doctor, or the institution? At the end of the day, it has to be the physician.” (Source: Interview 12—Resident, anesthesia)

4.2 Pedagogical and curricular needs: Participants emphasized the lack of formal AI education in current curricula, highlighted the need for ethical and critical appraisal skills when using AI tools, and consistently advocated for structured, hands-on workshops rather than purely didactic teaching. They called for:

- Formal AI education—Introducing AI topics in the curriculum.
- Ethical and critical appraisal training—Teaching how to critically evaluate AI outputs and understand ethical implications.
- Structured workshops and curricula—Practical training sessions and integrated modules on AI use.

“There was no touch upon AI at all in the curriculum, even though AI is already part of healthcare.” (Source: Interview 16—Intern)

“I don’t rely on it 100%… you need to recheck it to make sure everything is right. You need proper revision.” (Source: Interview 10—Resident)

“There should be workshops and hands-on training to teach us how to use AI properly, not just lectures.” (Source: Interview 8—Resident)

4.3 Barriers to adoption and readiness: Participants identified structural barriers to AI adoption, including limited access to resources, time pressures in clinical settings, institutional and policy-related restrictions, and concerns about patient trust when AI is visibly involved in care. Key barriers mentioned:

- Limited access and infrastructure—Not all necessary tools or data are readily accessible (e.g., paywalled guidelines).
- Time constraints—Clinicians have limited time, making it hard to incorporate AI unless it’s efficient.
- Institutional and policy restrictions—Organizational rules may limit AI use, and lack of institutional support can hinder adoption.
- Patient trust concerns—Worry that patients might trust clinicians less if AI is overtly used in care.

“Not all of the guidelines are free to use, so that’s why AI does not have access to them.” (Source: Interview 10—Resident)

“Sometimes I only have three to five minutes, so I need something quick, but I still have to recheck it.” (Source: Interview 12—Resident, anesthesia)

“If the institute or the government would provide verified tools for AI that we can use safely, that would make us more comfortable using AI.” (Source: Interview 16—Intern)

“If the patient knows this is a robot, it takes away a big part of the trust and the emotional connection.” (Source: Interview 6—Emergency medicine resident)

4.4 Humanistic boundaries and professional identity: Participants emphasized the irreplaceable role of human empathy, communication, and the clinician–patient relationship in healthcare. Across interviews, trainees consistently described AI as a supportive tool that should not replace the human elements of care.

Participants highlighted the importance of empathy and communication as core aspects of clinical practice, noting that emotional support and interpersonal interaction remain uniquely human strengths. They also emphasized the central role of the clinician–patient relationship in ensuring quality care and maintaining patient trust. In this context, AI was viewed as an assistive technology that complements, rather than replaces, human involvement in patient care.

These perspectives are illustrated in the following excerpts:

“When a patient is going through a hard time and you sit and speak to them, share what they have gone through, I’m not sure how AI would do this.” (Source: Interview 6—Emergency medicine resident)

“If the doctor uses AI in the wrong way, it can affect the relationship with the patient. You need to work on your communication skills, not act like an AI.” (Source: Interview 11—Medical student)

“AI is going to have a great impact on healthcare, but not to a point where it would replace humans.” (Source: Interview 3—Dental intern)

Open-ended survey responses reinforced these findings, with participants highlighting the importance of maintaining human-centered care alongside AI use. For example, one medical student noted the need for “how to properly and safely use it, including ethics,” emphasizing the importance of balancing technological use with professional responsibility.

Conceptual mind map: The relationships among these themes are visualized in a conceptual model illustrating how educational preparedness, ethical governance, and system-level factors collectively influence trainees’ trust and adoption of AI (Figure 1).

## Discussion—integration of quantitative and qualitative findings

This explanatory sequential mixed-methods study provides context-specific insights into how medical and dental trainees in the United Arab Emirates engage with and perceive artificial intelligence (AI) in healthcare. By integrating quantitative findings with qualitative interviews and open-ended survey responses, the study highlights a consistent pattern: trainees are actively using and valuing AI, yet their engagement remains largely informal, variably understood, and insufficiently supported by structured education and governance frameworks. The integration of findings across data sources is summarized in Table 2, which illustrates areas of convergence and divergence between quantitative trends, qualitative themes, and open-ended responses.

**TABLE 2:** Integrated qualitative-quantitative findings.

Quantitative finding (%, *n*)	Qualitative theme	Interview evidence	Open-ended survey evidence	Integrated interpretation
70.1% (108/154) reported no formal AI training	Educational gaps and need for structured training	“We need… a short training… especially for healthcare” Resident interview 10	“Need practice”; “Not fully explored”; “learning curve”	AI use is widespread but learning is informal and lacks structured guidance
High use of AI for academic tasks (frequent summarization, explanations)	AI as a tool for academic productivity and routine workflow	“I use ChatGPT… for studying and generating questions” Medical student interview 2	“Saving time”; “quick answers”; “easy summaries”	AI appears to be normalized as a routine academic support tool
∼76% agreement AI improves diagnostic accuracy	Perceived value and efficiency of AI	“It helps… find evidence-based knowledge quickly” Dental intern interview 3	“Fast and efficient”; “improves knowledge”	Trainees recognize strong practical benefits of AI
51.9% aware, 27.9% unsure of AI applications	Variable understanding of AI capabilities	“It depends on how you phrase the question” Medical student interview 11	“Not always accurate”; “depends on prompt”	Awareness exists but depth of understanding is inconsistent
61.0% feel prepared to use AI	Confidence gaps and reliance on experience	“I don’t know how to use it properly… just basic use” Urology resident interview 7	“Need practice”; “not fully confident”	Perceived preparedness is based on exposure, not structured competence
Limited recognition of indirect identifiers (∼53–64%)	Ethical concerns and data governance uncertainty	“You shouldn’t paste patient info… but not sure what exactly is safe” Interview 12	“Confidentiality concerns”; “data may leak”	Ethical awareness is partial, especially for indirect data risks
43.5% (67/154) prefer required AI training	Demand for structured and formal AI education	“There should be workshops… not just lectures” Interview 8	“Training needed”; “hands-on teaching”	Strong demand for structured and applied AI training
82.9% workshops, 70.4% simulations preferred	Preference for experiential learning approaches	“Workshops would help… practical training is better” Interview 1	“Hands-on sessions needed”	Trainees prefer applied, skill-based learning formats
High academic use vs. limited clinical use (e.g., 33.9% never use clinical AI tools)	Context-dependent trust in AI	“I use it for study… but not rely on it clinically” Interview 8	“Not fully reliable”; “need to verify”	Trust varies by task (academic vs. clinical)
45.6% residents, 63.6% interns unsure about AI functioning	Confidence gaps and limitations awareness	“AI can hallucinate… you must recheck everything” Interview 12	“Inaccurate”; “false information”; “bias”	Trainees recognize limitations but lack deeper understanding
Mixed views on job security (only ∼22% concerned)	Uncertainty about professional impact	“I don’t fully trust AI… still rely on books” Interview 8	“Cannot fully trust”; “needs human thinking”	No clear consensus on AI’s long-term professional impact

A central finding is the disconnect between widespread AI use and limited formal training. Quantitative results demonstrated that the majority of trainees had not received formal AI training, despite frequent use of AI tools, particularly for academic tasks. This pattern aligns with previous studies highlighting gaps in structured AI education within medical curricula (3, 7, 8). As shown in Table 2, this finding was reinforced by qualitative and open-ended data, where trainees described routine use of AI for summarization, question generation, and rapid information retrieval. Participants frequently highlighted benefits such as “saving time” and “quick answers,” suggesting that AI appears to be embedded in learning practices through informal and self-directed pathways, consistent with prior literature (9, 19). This aligns with findings from the UAE context, where university students demonstrate strong engagement with digital technologies and generally positive attitudes toward AI, despite variability in formal training and levels of eHealth literacy (20).

While survey data indicated generally positive attitudes toward AI, qualitative findings revealed a more nuanced and conditional engagement. As summarized in Table 2, there is clear convergence between positive perceptions of AI’s utility and qualitative descriptions of efficiency and productivity gains. However, divergence is evident in relation to trust. Trainees expressed confidence in AI for low-stakes academic tasks but emphasized caution in clinical contexts. This pattern reflects findings in the literature on context-dependent trust in AI, where acceptance varies by perceived risk (6, 10). Interview and open-ended data highlighted concerns about inaccuracy, bias, and unreliable sources, suggesting that while AI is valued, it may not be fully trusted. This suggests the added value of qualitative data in revealing underlying uncertainty not fully captured in survey responses.

The study also identified variation in perceptions across training levels, although these should be interpreted as descriptive patterns. Interns and medical students reported higher expectations for AI applications, whereas residents demonstrated more cautious perspectives. As shown in Table 2, qualitative findings provide context for this pattern, suggesting that earlier-stage trainees engage with AI primarily as a learning tool, while residents emphasize clinical responsibility, accountability, and real-world limitations. This aligns with previous studies suggesting that increased clinical exposure is associated with more critical appraisal of AI tools (6, 11).

Another important finding relates to perceived preparedness and skill gaps. Although many trainees reported feeling prepared, the integrated findings (Table 2) reveal that this preparedness is often based on informal experience rather than structured competency development. Qualitative data highlighted the need for skills such as prompt formulation, critical evaluation, and understanding of AI limitations. These findings are consistent with literature emphasizing that effective AI use may require specific competencies, including interpretability and critical appraisal (5, 6, 8). This highlights a gap between perceived readiness and actual capability, highlighting the potential value of competency-based education.

The findings also reveal a mixed landscape of ethical awareness. While quantitative data indicated strong recognition of direct privacy risks, Table 2 suggests divergence in the understanding of indirect identifiers and data governance. Qualitative and open-ended responses highlighted uncertainty regarding confidentiality and safe data-sharing practices, alongside concerns about bias and reliability. These findings align with existing literature identifying gaps in ethical understanding among healthcare professionals (3, 5) and highlight the potential value of explicit education in ethical and governance aspects of AI.

A further key insight is the context-dependent nature of trust in AI, consistently observed across all data sources. As illustrated in Table 2, trainees reported high trust in AI for academic tasks but demonstrated caution in clinical decision-making. This aligns with literature showing that adoption depends on perceived reliability, explainability, and workflow integration (10, 21). The consistent emphasis on verification and human oversight suggests that trainees view AI as an assistive rather than autonomous tool, supporting the continued importance of clinical judgment.

Beyond individual competencies, trainees framed AI integration as a system-level issue, emphasizing the role of institutions in providing validated tools, structured training, and governance frameworks. As reflected in Table 2, there is strong convergence between quantitative preferences for structured training and qualitative calls for workshops and hands-on learning. These findings align with recommendations for experiential and applied AI education (7, 8, 21) and suggest that effective integration may benefit from a socio-technical approach.

Taken together, findings from this UAE-based sample suggest that AI integration in healthcare training may benefit from longitudinal, clinically relevant, and ethically grounded educational strategies, supported by institutional and policy-level frameworks. However, given the descriptive design and context-specific setting, these findings should be interpreted as exploratory. The consistency across quantitative, qualitative, and open-ended data summarized in Table 2 provides support for the overall patterns observed and highlights the value of mixed-methods approaches in understanding complex educational phenomena. These findings should be interpreted within the context of a single national setting and descriptive analysis.

The authors’ use of AI tools for language support, with full human oversight and without data exposure, reflects the importance of responsible and transparent AI use in academic practice, consistent with the themes of critical appraisal and ethical awareness identified among trainees.

### Strengths

This study has several important strengths. First, the use of an explanatory sequential mixed-methods design enabled both the identification of patterns through quantitative data and the in-depth explanation of these patterns through qualitative findings. The integration of survey results, interview data, and open-ended responses summarized through a joint display enhanced interpretive validity by demonstrating convergence and divergence across data sources.

Second, the inclusion of trainees across multiple stages of the training continuum (medical students, interns, and residents) provides a comprehensive perspective on AI readiness and highlights how engagement with AI evolves with clinical exposure. This multi-level approach offers insights relevant to the design of longitudinal and stage-specific educational interventions.

Third, the study contributes context-specific empirical evidence from a Middle Eastern healthcare setting, addressing a gap in a literature that is largely dominated by Western perspectives. The inclusion of participants from multiple emirates enhances the relevance of findings within similar healthcare systems undergoing digital transformation.

Finally, the qualitative component was conducted with methodological rigor, including the use of reflexive thematic analysis following Braun and Clarke’s framework, adherence to COREQ reporting standards (22), and the incorporation of illustrative participant quotations. These approaches support the credibility, transparency, and depth of the findings.

### Limitations

Several limitations should be considered when interpreting these findings. First, the study employed a convenience sampling approach with voluntary participation, which may introduce selection bias, as trainees with greater interest in or exposure to artificial intelligence may have been more likely to respond. The overall sample size, while adequate for descriptive analysis, included unequal subgroup distributions, with relatively small numbers of interns and medical students compared to residents. As a result, findings related to differences across training levels should be interpreted cautiously.

Second, quantitative data were analyzed using descriptive statistics only, and no inferential statistical testing was performed. This approach was appropriate given the exploratory design, modest sample size, and subgroup imbalance; however, it limits the ability to draw conclusions about statistically significant differences or generalize findings beyond the study sample.

Third, all quantitative measures were based on self-reported data, which may not accurately reflect actual knowledge, competence, or real-world behavior. Perceived preparedness and AI use may therefore be overestimated or underestimated. In addition, key constructs such as knowledge, preparedness, and attitudes toward AI are inherently complex and multifaceted, and their assessment using self-reported survey items may provide only an approximate representation of trainees’ actual competencies, behaviors, and understanding. Accordingly, findings related to these domains should be interpreted with caution.

In addition, although the questionnaire was informed by existing literature and refined through expert review, the absence of formal pilot testing or cognitive interviewing may limit the ability to fully assess item interpretation and response consistency.

In addition, the cross-sectional design of the survey component precludes conclusions about how trainees’ attitudes, knowledge, and practices evolve over time, highlighting the need for longitudinal research to better understand changes across training stages.

Fourth, the qualitative sample size (*n* = 16), while sufficient for thematic depth and supported by the concept of information power, may not capture the full range of trainee perspectives, particularly among those with limited engagement with AI or more critical viewpoints. Additionally, open-ended survey responses were used primarily to support triangulation rather than as a standalone qualitative dataset, which may limit the breadth of qualitative insight.

Finally, the study was conducted within a single national context (UAE), which may limit generalizability to other healthcare systems. However, the core patterns identified particularly regarding trust in AI, gaps in formal education, and the importance of governance are consistent with findings reported in international literature (6, 19), suggesting broader relevance.

### Future directions

Future research should focus on evaluating the impact of structured, competency-based AI education on trainees’ knowledge, confidence, and clinical decision-making across different stages of training. While interest in AI education is well documented, empirical evidence on the effectiveness of specific curricular interventions remains limited (7, 19). Longitudinal studies following trainees from undergraduate education through residency are particularly needed to understand how AI readiness evolves over time and to identify the optimal timing, depth, and sequencing of AI-related competencies within medical curricula (6).

In addition, comparative interventional studies should assess the effectiveness of hands-on, workflow-integrated educational approaches, such as simulations, clinical use cases, and applied problem-based learning, in developing practical AI skills. These approaches may be more effective than traditional didactic methods in supporting safe, critical, and context-aware use of AI tools (7, 23).

Beyond individual-level training, future research should examine systems-level factors influencing AI adoption in healthcare education and practice. This includes evaluating how institutional policies, access to validated AI tools, IT infrastructure, and governance frameworks shape trainee trust, engagement, and responsible use of AI (10, 11). Understanding these socio-technical factors will be critical for designing scalable and sustainable AI integration strategies.

Finally, further investigation is needed into the broader implications of AI for professional identity, clinical reasoning, and the clinician–patient relationship. As AI becomes increasingly embedded in healthcare, it is essential to ensure that its integration supports, rather than undermines, human-centered care, empathy, and professional accountability (6, 9).

## Conclusion

This mixed-methods study provides descriptive, context-specific insights into how medical and dental trainees in the UAE engage with and perceive artificial intelligence in healthcare. Trainees reported widespread use of AI, particularly for academic purposes, alongside strong interest in structured education.

At the same time, findings suggest important gaps in formal training, variability in preparedness, and incomplete understanding of ethical and data-related considerations. Trainees showed context-dependent trust in AI, emphasizing its role as an assistive tool rather than a replacement for clinical judgment.

These findings suggest that effective integration of AI into medical education may benefit from structured, clinically relevant, and ethically grounded approaches within institutional and governance frameworks. While exploratory in nature, the study provides preliminary insights to inform future research and curriculum development aimed at supporting trainees’ preparedness for AI-enabled healthcare.

## Data Availability

The datasets presented in this study can be found in online repositories. The names of the repository/repositories and accession number(s) can be found in this article/Supplementary material.

## Supplementary material

Supplementary Data 1 Survey questionnaire.

Supplementary Data 2 Interview guide.

Supplementary Data 3 COREQ checklist.
