# Supplementary material for: Preparing tomorrow’s physicians for AI-driven healthcare: insights from a study on medical students’, interns’, and residents’ knowledge, attitudes, and educational needs
Source: Front Med (Lausanne). 2026 Jun 2;13:1799061. doi: 10.3389/fmed.2026.1799061 (PMC13269010; doi:10.3389/fmed.2026.1799061)
Supplement: Supplementary file 1 [file Data_Sheet_4.pdf]

Supplementary Data 4: Expanded Theme Table

Description: This supplementary table provides an expanded overview of the qualitative thematic analysis conducted in this study. It outlines the main themes, sub-themes, and sub-sub-themes, along with illustrative quotations from semi-structured interviews and selected open-ended survey responses. Interview data served as the primary qualitative source, while survey comments were used selectively to corroborate emerging themes, consistent with the triangulation approach described in the Methods section. Quotations are anonymized and labeled by trainee type to preserve confidentiality

Main Theme	Sub-Theme	Key codes under Sub-Themes	Illustrative Quote (Verbatim)	Data Source	Trainee Type
**Perceived Value and Normalization of AI**	Optimism and enthusiasm toward AI	AI as efficient and transformative; Time-saving and workload reduction	“AI is going to have a great impact on healthcare. It will make our work easier and faster, but it should be used in the right way.”	Interview	Dental Intern
	Routine use for academic productivity	Daily use; Studying and exam preparation	“I used it to generate several questions so that I can train myself for the question material when studying for exams.”	Interview	Medical Student
	AI in research and scholarly work	Research idea generation; Literature summarization; Writing support	“AI makes things faster, but I still have to recheck everything before I use it.”	Interview	Resident
**AI as a Tool for Clinical Support and Workflow Optimization**	Clinical decision support	Radiology and imaging support; Triage and prioritization	“With X-rays and CTs they have implemented AI… it helps the assessment to be quicker and more efficient.”	Interview	Resident
	Workflow efficiency	Reduced documentation burden; Improved patient care	“AI can help with routine stuff, for example writing notes… so the doctor can focus more on the patient.”	Interview	Intern
	Administrative and operational support	Scheduling; Task organization	“We used it to do the on-call rota… it’s a headache for a human being.”	Interview	Resident
**Trust, Confidence, and Human Oversight**	Confidence gaps and perceived limitations	Accuracy concerns; Bias; Reliability issues	“AI will state the answer in a very professional way… but the knowledge is wrong.”	Interview	Resident
	Context-dependent trust	High trust for low-stakes tasks; Low trust for clinical decisions	“I can rely on AI for studying, but not for treatment decisions.”	Interview	Intern
	Human oversight and responsibility	Cross-checking; Professional judgment	“I don’t rely on it fully… I still have to recheck after.”	Interview	Resident

**Ethical, Educational, and System-Level Preconditions**	Ethical and privacy concerns	Data security; Confidentiality	“You shouldn’t copy patient information into AI tools because it’s private.”	Interview	Resident
	Educational needs	Formal AI education; Ethical appraisal training	“There was no touch upon AI at all in the curriculum.”	Interview	Intern
	Institutional and policy readiness	Governance; Validated AI tools; IT infrastructure	“If the institute or government provides verified tools, we would feel more comfortable using AI.”	Interview	Intern
	Humanistic boundaries	Empathy; Clinician–patient relationship	“A robot can never show empathy like a human can.”	Interview	Resident
**Triangulation with Survey Data**	Reinforcement of themes	Efficiency; Ethics; Trust	“Smoother workflow and less paperwork.”	Survey	Medical Student
