# Supplementary material for: Preparing tomorrow’s physicians for AI-driven healthcare: insights from a study on medical students’, interns’, and residents’ knowledge, attitudes, and educational needs
Source: Front Med (Lausanne). 2026 Jun 2;13:1799061. doi: 10.3389/fmed.2026.1799061 (PMC13269010; doi:10.3389/fmed.2026.1799061)
Supplement: Supplementary Data 1 — Survey questionnaire. [file Data_Sheet_1.pdf]

Preparing Tomorrow's Physicians for AI-Driven Healthcare

Age

____

What is your level of experience with artificial intelligence (AI)?

- ◯ None
- ◯ Basic
- ◯ Intermediate
- ◯ Advanced

Have you attended any AI-related training or education programs?

- ◯ Yes - Formal
- ◯ Yes - Informal
- ◯ No

Knowledge About AI in Healthcare

What is the best definition of Artificial Intelligence (AI)?

- ◯ A robot that performs surgery
- ◯ Computer systems that simulate human intelligence
- ◯ Software for hospital logistics
- ◯ Not sure

Are you aware of the applications of AI in healthcare?

- ◯ Yes
- ◯ No
- ◯ Not Sure

What are the applications of AI in healthcare? (Select all that apply)

- ☐ Diagnosing diseases
- ☐ Predicting outbreaks
- ☐ Automating documentation
- ☐ Replacing doctors completely

AI reduces Medical Errors

- ◯ True
- ◯ False
- ◯ Not sure

AI decisions are always unbiased.

- ◯ True
- ◯ False
- ◯ Not sure

AI tools need large data to work.

- ◯ True
- ◯ False
- ◯ Not sure

Attitudes Toward AI in Medicine

Please rate from 1 (Strongly Disagree) to 5 (Strongly Agree):

	Strongly Disagree	Disagree	Neutral	Agree	Strongly Agree
AI will improve diagnostic accuracy	◯	◯	◯	◯	◯

I understand how AI works in clinical settings	◯	◯	◯	◯	◯
AI threatens physicians' job security	◯	◯	◯	◯	◯
AI cannot replace human empathy	◯	◯	◯	◯	◯
AI should be part of medical training	◯	◯	◯	◯	◯
I'm interested in learning more about AI	◯	◯	◯	◯	◯
AI raises important ethical concerns	◯	◯	◯	◯	◯
Doctors should be involved in AI development	◯	◯	◯	◯	◯

Educational Needs and Preferences

Do you feel prepared to work with AI in healthcare?

- ◯ Yes
- ◯ No
- ◯ Not Sure

Would you like formal AI training in your curriculum?

- ◯ Required
- ◯ Elective
- ◯ Workshop
- ◯ No

Which of the following topics are you interested in? (select all that apply)

- ☐ Machine learning basics
- ☐ Ethics and law
- ☐ Clinical use cases
- ☐ Evaluating AI tools
- ☐ Other, please specify

If Others, please specify

____

What are your preferred learning formats? (choose up to two)

- ☐ Lectures
- ☐ Online modules
- ☐ Workshops
- ☐ Guest speakers
- ☐ Simulations
- ☐ Not interested

Experience with AI in Education or Clinical Practice

Please indicate whether you have used AI tools or applications in the following areas.
For each item, select the frequency that best describes your experience:
(Options: 1. Never 2. Rarely 3. Sometimes 4. Often 5. Very Often)

	Never	Rarely	Sometimes	Often	Very Often
Practicing Multiple Choice Questions (MCQs) with AI-driven tools (e.g., question generators, adaptive quizzing)	○	○	○	○	○
Writing essays or assignments with help from AI (e.g., ChatGPT, Copilot)	○	○	○	○	○
Using AI tools to study or summarize medical content	○	○	○	○	○
Generating explanations or understanding complex topics with AI assistants	○	○	○	○	○
Using AI to manage your academic or clinical schedule	○	○	○	○	○
Using AI-assisted differential diagnosis or investigation suggestions	○	○	○	○	○
Exposure to AI-supported diagnostic tools during rotations (e.g., AI in radiology, dermatology)	○	○	○	○	○
Clinical decision support systems incorporating AI (e.g., electronic prescribing, risk prediction)	○	○	○	○	○

Please describe any specific AI tools or platforms you've personally used in your medical education or clinical practice

____

What benefits or challenges have you experienced when using AI in your learning or practice?

____

Which of the following should NOT be shared with general AI tools? (Select all that apply)

☐ Patient name
☐ Patient ID
☐ Clinical notes with identifiers
☐ Imaging data with metadata
☐ None of the above

Has the introduction of AI tools changed the way you study or approach clinical practice?

○ Yes - significantly
○ Yes - somewhat
○ No - not really
○ Not sure

If yes, please explain how AI has changed your study habits or clinical workflow:

____

Career Implications	
Will AI influence your specialty choice?	◯ Yes - I prefer an AI-integrated specialty<br>◯ Yes - I will avoid AI-heavy specialties<br>◯ No impact<br>◯ Not sure
What type of career do you envision?	◯ Clinical<br>◯ Clinical + academic<br>◯ Academic/research<br>◯ Not sure
Open Ended Comments	
What excites or concerns you most about AI in healthcare?	______________________________
What should medical schools or training programs teach about AI?	______________________________
What challenges do you foresee in learning or using AI in your career?	______________________________
Have you encountered AI tools in clinical work? Describe.	______________________________
Do you feel your training level has prepared you for AI integration? Explain.	______________________________
If you're not interested in AI, why?	______________________________
Would you join a follow-up interview or focus group?	◯ Yes<br>◯ No
If Yes, please share the email	________________________
If Yes, please share the Mobile	________________________
