# Supplementary material for: Preparing tomorrow’s physicians for AI-driven healthcare: insights from a study on medical students’, interns’, and residents’ knowledge, attitudes, and educational needs
Source: Front Med (Lausanne). 2026 Jun 2;13:1799061. doi: 10.3389/fmed.2026.1799061 (PMC13269010; doi:10.3389/fmed.2026.1799061)
Supplement: Supplementary Data 2 — Interview guide. [file Data_Sheet_2.pdf]

Post-Survey Focus-Group Interview Guide

Purpose: Explore participants' real-world experiences, needs, and perceptions of AI in healthcare, building on our survey findings.

---

1. **Current Experience & Usage**
2. **Competency & Confidence**
3. **Educational Preferences & Gaps**
4. **Ethical, Legal & Organizational Context**
5. **5. Role-Specific Implementation**

Below, I map each domain to a handful of sharp, interview-ready questions—pruned from your draft—and suggest precise wording to elicit the deepest possible responses.

---

1. Current Experience & Usage

Goal: Document exactly how and when learners actually interact with AI tools, so you can describe real-world usage patterns.

- **"Tell me about the last time you used an AI tool during your studies or clinical work. What was the context and what steps did you take?"**
- **"Have you ever wanted to use an AI application but weren't able to? What stopped you?"**
- **What AI application did use and was it accessed for free or via a paid license (personally or through your institution)?**

---

2. Competency & Confidence

Goal: Gauge self-assessed ability and comfort interpreting or validating AI outputs.

- **“On a scale from 1–5, how confident do you feel evaluating AI recommendations? Can you give an example of when you felt under- or over-confident?”**
- **“What specific skills—if any—would make you feel more prepared to work with AI?”**

---

3. Educational Preferences & Gaps

Goal: Pin down exactly which instructional formats and content areas learners want, and what they feel is missing.

- **“What format (lecture, hands-on workshop, simulation, etc.) would help you best learn to use AI? Why?”**
- **“Which AI topics do you think are completely missing from your curriculum today?”**

---

4. Ethical, Legal & Organizational Context

Goal: Surface concrete concerns, real or anticipated, that will shape policy and support needs.

- **“Can you share a situation where you worried about data privacy or algorithmic bias? What happened and how did you respond?”**
- **“What institutional supports (guidelines, supervision, tech infrastructure) would make you comfortable adopting AI in your workflow?”**

---

5. Role-Specific Implementation

A. Medical Students

1. **Rotation Preparedness**
    - How prepared do you feel to interpret AI outputs during clinical rotations? Why?
2. **Hands-On Experience**

- Which AI tools or platforms have you personally used in your education or practice? What benefits or challenges did you experience?

B. Interns & Residents

1. **First Steps**
    - If you were tasked with implementing AI in your hospital, what would your first step be?
2. **Task Allocation**
    - What clinical tasks do you think AI should—and should not—be used for?
3. **Evolving Relationships**
    - How do you see the doctor–patient relationship evolving in a more AI-driven environment?

Interview Tips:

- **Probe for Examples:** Always ask "Can you give me an example?" to ground abstract ideas in experience.
- **Follow Up:** If a response is brief, use prompts like "Tell me more about that…" or "What led you to feel that way?"
- **Time Management:** Aim for ~45–60 minutes total; you may skip or shorten lower-priority items if time runs short.
- **Concise & Focused:** Each probes one construct deeply, avoiding double-barreled or leading phrasing.
- **Behavioral Anchors:** Asking for "last time" or "concrete example" unearths stories rather than abstractions.
- **Actionable Outputs:** The combination of scale+example, preference+rationale, and gap-identification delivers both vision and practical detail.

Methodology

1. Record via Microsoft Teams

- **Schedule & Launch**
  - Start your focus-group as a Teams meeting.
- **Built-In Recording**
  - Hit "Record"—Teams captures audio (and video) and stores it in your org's Stream/OneDrive.
- **Auto-Transcription**
  - Enable "Allow transcription" before you start. When the meeting ends, Teams will generate a time-stamped transcript (VTT/Word) automatically.

---

2. Clean & Prepare the Transcript

1. **Download** the VTT or DOCX transcript from Teams.
2. **Quick Edit** in Word:
   - Fix speaker labels (if needed)
   - Remove filler ("um", "ah") for readability

---

3. Initial Coding & Mapping

- **Taguette** (free, browser-based)
  1. Import your cleaned transcript.
  2. Create codes (e.g., "Trust," "Workflow Barriers," "Training Needs").
  3. Tag relevant passages.
- **Microsoft OneNote** (if you prefer staying in the MS ecosystem)
  - Paste transcript, highlight sections in different colors, and add tags/comments.

---

4. AI-Assisted Thematic Analysis with ChatGPT Team

Used paid version of ChatGPT, you can batch-process larger text directly and keep everything private under your org:

1. **Chunk & Summarize**
   - Paste ~1–2 pages of transcript into ChatGPT with:

“Using the coding framework [list your codes], summarize each code’s key themes and pull two illustrative quotes.”

2. **Refine & Expand**
   - Ask follow-ups like:

“What sub-themes emerge under ‘Data Privacy’?”

3. **Generate a Codebook**
   - Prompt:

“Produce a table: Code | Definition | Example Quote.”

   - Copy into Excel or Google Sheets for formatting.

---

5. Visual Mapping

- **Free Miro (or Microsoft Whiteboard)**
  1. Create nodes for each major theme.
  2. Drag in representative quotes or sub-themes.
  3. Connect related nodes to show overlaps.
- **Alternative: Coggle**
  - Quick, shareable mind-maps you can export as PNG/SVG for your report.

---

6. (Optional) Lightweight Quantitative Check

If you want simple frequency counts of keywords/themes:

- **Excel or Google Sheets**

    - Paste your tagged transcript, add a column for “Code,” then use pivot tables to count occurrences.
- **Python Script (local)**
    - Run a 10–15 line script with spaCy or NLTK to tally code frequencies over the interview timeline.

Workflow Recap

1. **Teams** → record + transcript
2. **Clean transcript** → Taguette/OneNote coding
3. **ChatGPT Team** → thematic summaries & codebook
4. **Miro/Whiteboard** → visual theme map
5. **Excel/Script** → simple frequency check

All tools either come with your MS 365 subscription or are free, leverages your existing, and injects AI where it accelerates (the transcription and thematic distillation).
