# Supplementary material for: Preparing tomorrow’s physicians for AI-driven healthcare: insights from a study on medical students’, interns’, and residents’ knowledge, attitudes, and educational needs
Source: Front Med (Lausanne). 2026 Jun 2;13:1799061. doi: 10.3389/fmed.2026.1799061 (PMC13269010; doi:10.3389/fmed.2026.1799061)
Supplement: Supplementary Data 3 — COREQ checklist. [file Data_Sheet_3.pdf]

COREQ (COnsolidated criteria for REporting Qualitative resea

A checklist of items that should be included in reports of qualitative research. You must report the page number in your manuscript where you consider each of the items listed in this checklist. If you have not included this information, either revise your manuscript accordir

Topic	Item No.	Guide Questions/Description
**Domain 1: Research team and reflexivity**		
Personal characteristics		
Interviewer/facilitator	1	Which author/s conducted the interview or focus group?
Credentials	2	What were the researcher’s credentials? E.g. PhD, MD
Occupation	3	What was their occupation at the time of the study?
Gender	4	Was the researcher male or female?
Experience and training	5	What experience or training did the researcher have?
Relationship with participants		
Relationship established	6	Was a relationship established prior to study commencement?
Participant knowledge of the interviewer	7	What did the participants know about the researcher? e.g. personal goals, reasons for doing the research
Interviewer characteristics	8	What characteristics were reported about the inter viewer/facilitator?
**Domain 2: Study design**		
Theoretical framework		
Methodological orientation and Theory	9	What methodological orientation was stated to underpin the study? e.g. grounded theory, discourse analysis, ethnography, phenomenology, content analysis
Participant selection		
Sampling	10	How were participants selected? e.g. purposive, convenience, consecutive, snowball
Method of approach	11	How were participants approached? e.g. face-to-face, telephone, mail, [illegible]
Sample size	12	How many participants were in the study?
Non-participation	13	How many people refused to participate or dropped out? Reasons?
Setting		

Setting of data collection	14	Where was the data collected? e.g. home, clinic, workplace
Presence of non-participants	15	Was anyone else present besides the participants and researchers?
Description of sample	16	What are the important characteristics of the sample? e.g. demographic data, date
Data collection		
Interview guide	17	Were questions, prompts, guides provided by the authors? Was it pilot tested?
Repeat interviews	18	Were repeat interviews carried out? If yes, how many?
Audio/visual recording	19	Did the research use audio or visual recording to collect the data?
Field notes	20	Were field notes made during and/or after the interview or focus group?
Duration	21	What was the duration of the inter views or focus group?
Data saturation	22	Was data saturation discussed?
Transcripts returned	23	Were transcripts returned to participants for comment and/or correction?
**Domain 3: analysis and findings**		

Data analysis		
Number of data coders	24	How many data coders coded the data?
Description of the coding tree	25	Did authors provide a description of the coding tree?
Derivation of themes	26	Were themes identified in advance or derived from the data?
Software	27	What software, if applicable, was used to manage the data?
Participant checking	28	Did participants provide feedback on the findings?
Reporting		
Quotations presented	29	Were participant quotations presented to illustrate the themes/findings? Was each quotation identified? e.g. participant number
Data and findings consistent	30	Was there consistency between the data presented and the findings?
Clarity of major themes	31	Were major themes clearly presented in the findings?
Clarity of minor themes	32	Is there a description of diverse cases or discussion of minor themes?

Developed from: Tong A, Sainsbury P, Craig J. Consolidated criteria for reporting qualitative research (COREQ): a 32-item checklist for interviews and focus groups. *International Journal for Quality in Health Care* . 2007. Volume 19, Number 6: pp. 349 – 357

Once you have completed this checklist, please save a copy and upload it as part of your submission. DO NOT include this checklist as part of the main manuscript document. It must be uploaded as a separate file.

rch) Checklist

ngly before submitting or note N/A.

Reported on Page No.
Dr. Asma Syeda/Fatima Alriyami/ Maha Alketbi/Asma Alshbli
PhD/MBBS/MBBS/MBBS
Faculty/Medical Students
Female
Researcher have publications from past researches
Yes
Yes
Yes
[illegible] methodological orientation, using inductive reflexive thematic analysis as
A convenience sampling approach was used for survey recruitment. Interview
Particpants were invited to an Online survey via email and those who agreed via
A total of 150 responded to the survey out of which 35 agreed to participate initially but eventually only 16 were available for the interviews
**19**

The survey was conducted online & interviews were conudcted via teams. The interviews recorded and transcribed. Both recordings & transcripts were archived in dedicated folders. The researchers divided the indivula interviews equally and listened to the recordings to vlaidate the transcripts. The edited versions of the transcripts were also archived in a separate folder.
Yes, The Facilitator, notetaker and the participants
There were 79.53% Females & 20.47% Male trainees who responded to the Survey. The participants constituted three groups, firstly residents who were post graduate medical education trainees from medical & dental programs (62.43%), secondly Interns who are currently enrolled in the medical & dental internship programs across UAE (12.13%) and thirdly medical students from UAEU across all years (25.44%). The mean age of the candidates was 28.75. Interview participants: A total of 16 participants took part in the interviews, of whom 68.75% were female and 31.25% were male. The interview cohort comprised three groups: postgraduate residents from medical and dental programs (62.50%), representing different stages of training (R2–R5, including final-year residents and a recently completed resident); interns enrolled in medical and dental
Yes
Yes
Yes, audio . Interviews done via Teams
The interview was conducted via Teams and captured all comments (video recording as well as transcripts) from particpants and the researchers had list of details of the participants
minutes and highest duration of 50 minutes.
Yes
**Yes**

4 (FOUR)
Yes
Themes were identified in advance
NVivo, LLM, Microsoft (word, excel, teams), Notebook LM, SPSS,
**Yes**
**Yes**
**Yes**
**Yes**
**Yes**
